# Recent advances in understanding and managing non-alcoholic fatty liver disease

**DOI:** 10.12688/f1000research.14421.1

**Published:** 2018-06-11

**Authors:** Somaya Albhaisi, Arun Sanyal

**Affiliations:** 1 The Division of Internal Medicine, Virginia Commonwealth University, Richmond, USA; 2 The Division of Gastroenterology, Hepatology and Nutrition, Virginia Commonwealth University, Richmond, USA

**Keywords:** Nonalcoholic fatty liver disease, Nonalcoholic steatohepatitis, cirrhosis, fibrosis

## Abstract

Non-alcoholic fatty liver disease is a leading cause of chronic liver disease that can lead to cirrhosis, hepatocellular cancer, and end-stage liver disease, and it is linked to elevated cardiovascular- and cancer-related morbidity and mortality. Insulin resistance related to metabolic syndrome is the main pathogenic trigger that, in association with adverse genetic, lifestyle, and other factors, precipitates the development of non-alcoholic fatty liver disease. Biochemical markers and radiological imaging, along with liver biopsy in selected cases, help in the disease’s diagnosis and prognostication. Weight loss is the cornerstone treatment of non-alcoholic fatty liver disease; however, it is difficult to achieve and maintain, so pharmacotherapy was developed. The remarkable evolution in understanding disease pathogenesis has led to the development of new medical therapies and even the modification of currently available ones. This review summarizes recent advances in understanding the epidemiology, natural history, pathogenesis, diagnosis, and management of non-alcoholic fatty liver disease.

## Introduction

Non-alcoholic fatty liver disease (NAFLD) is the condition in which hepatic fat accumulation is present after all other causes of hepatic steatosis are excluded; these include liver disease caused by other factors, excessive alcohol consumption, and other conditions that may lead to hepatic steatosis. The clinical spectrum of NAFLD is wide-ranging and spans NAFL to non-alcoholic steatohepatitis (NASH), advanced fibrosis, cirrhosis, and hepatocellular carcinoma (HCC). NAFLD is the most common liver disease in the world, and NASH may soon become the most common indication for liver transplantation ^1^. The incidence and prevalence of NAFLD is rising globally owing to increasing rates of obesity and diabetes ^2, 3^. The development of liver cancer in patients with NAFLD, even without the presence of cirrhosis, was observed recently in a number of studies. These outcomes contribute substantially to the burden of disease to the individual and also to society. It is therefore a public health priority to develop effective measures for the identification and treatment of this condition. The recent advances in understanding and managing NAFLD are reviewed below.

## Epidemiology

The true worldwide incidence rate of NAFLD/NASH is not known. It was found through a few studies that the incidence rates were widely variable owing to multiple factors which include the different characteristics of study populations, exclusion criteria, study methodology, and approach for diagnosis. The variable presentations of the disease as well as the unavailability of sensitive diagnostic studies besides the liver biopsy, which remains the gold standard to date, probably contribute to the underreported incidence and prevalence of NAFLD ^4^. Analysis of liver ultrasound data collected between 1988 and 1994 from the Third National Health and Nutrition Examination Survey (NHANES III) reported that 19% of adults have NAFLD, while a meta‐analysis of studies from 2006–2014 estimated a NAFLD prevalence of 24% (20–29%) in the general population. The Dionysos ^5^ study in Italy first reported that the global prevalence of NAFLD is 24–25% of the general population; this is the most accurate estimated figure to date, and it was confirmed recently by Younossi ^6, 7^, who described some regional differences with the highest rates reported in South America and the Middle East, followed by Asia, the USA, and Europe. The increasing prevalence of NAFLD/NASH is in parallel to the pandemic spread of obesity, diabetes mellitus (DM), and metabolic syndrome. One-third of American adults are thought to have NAFLD ^8^. The prevalence of NAFLD in Europe and the Middle East ranges from 20–30%. The highest prevalence of NAFLD was reported in South America and the Middle East, whereas the lowest was reported in Africa. Although the global burden of NAFLD is unknown, on the basis of the above studies, we estimate that ~one billion individuals have NAFLD at the current time. Liver biopsy is the gold-standard diagnostic test. There are also some non-invasive diagnostic modalities including hepatic ultrasonography, computed tomography (CT), and magnetic resonance imaging (MRI). The discrepancy in prevalence data for NAFLD could be attributed to the difference in sensitivity of these diagnostic tests. Younossi *et al*. reported that the pooled regional NAFLD prevalence estimates among patients diagnosed by blood test were 13.00% (95% confidence interval [CI]: 4.44–32.47) for Europe, 12.89% (95% CI: 8.32–19.44) for North America, and 9.26% (95% CI: 7.07–12.05) for Asia ^9^. Estes *et al*. reported that by 2030, the NAFLD population was projected to increase by 21% to 100.9 million cases. Prevalence in 2030 is estimated at 33.5% (aged ≥15 years) and 28.4% (all ages). The NAFLD population is estimated to have a median age of 50 (2015), which increases to 55 by 2030. During 2015–2030, there would be 1.2 prevalent NAFLD cases among male individuals for every 1.0 among female individuals ^10^. Because of the small number of studies that contained NAFLD incidence results, Younossi *et al*.’s meta-analysis results were obtained only for Asia (only available for China and Japan) and Israel. In Asia, the pooled regional NAFLD incidence rate was estimated to be 52.34 per 1,000 person-years (95% CI: 28.31–96.77), and in Israel it was estimated to be 28.01 per 1,000 person-years (95% CI: 19.34–40.57) ^9^. According to Estes *et al*., the fastest growth in obesity prevalence occurred during 2000–2002. In comparison, the fastest growth in DM prevalence occurred during 2012–2014. The number of NAFLD cases increased following obesity, resulting in a peak NAFLD incidence in 2008 with an estimated 4.17 million new cases. Since then, a slowing rate of increase in NAFLD was forecasted, and new cases were estimated to decline to 3.62 million annually. Thus, the total number of NAFLD cases is still increasing but at a lower rate compared to the 2005–2008 period. However, given the rising prevalence of type 2 diabetes, the proportion of individuals with NASH is predicted to increase, and with prolonged disease exposure the number of individuals with end-stage liver disease is likely to triple by 2030. It has been estimated that, in the USA, NAFLD is responsible for $292 billion in annual medical and societal costs ^10^. The projected cost of caring for patients is expected to increase by 18% from 2000 to 2035, and the health-related quality of life of NAFLD patients is described as declining ^11, 12^.

## Pathogenesis

A substantial body of literature has accumulated and provides a framework to understand the pathogenesis of NAFLD. With increasing caloric intake and changes in dietary composition, excess calories are stored as fat within adipose tissue and also induce changes in the microbiome. This triggers changes in intestinal permeability and increased systemic exposure to intestinal microbial products, triggering activation of the innate immune system and adipose tissue inflammation. The metabolic consequence is the development of an insulin-resistant state. The insulin-resistant state which drives increased lipolysis along with the consumption of excess calories deliver an increased load of lipotoxic lipids including free fatty acids to the liver along with excess carbohydrates. This is further compounded by increased *de novo* lipogenesis, which is driven by hyperinsulinemia and retained sensitivity to the lipogenic effects of insulin in an otherwise insulin-resistant state. The liver attempts to react by increasing lipid oxidation and export of lipids; when lipid influx and synthesis exceed its metabolism and export, the excess lipids accumulate in lipid droplets, creating a fatty liver. Recently, it has been shown that the PNPLA3 protein accumulates on the surface of lipid droplets ^13^. Under conditions of lipotoxic stress, proteasomal impairment drives such accumulation where, in those with mutant PNPLA3, impaired lipolysis leads to further accumulation of fat. However, this does not explain how the mutation drives the development of steatohepatitis and cirrhosis. Cell stress including oxidative stress and unfolded protein response can trigger apoptosis, cell death, and inflammation. Apoptosis can also trigger cell regenerative activity. While much is known about how lipotoxicity drives cell death and inflammation, there is still a paucity of information on the biological mechanisms driving tissue adaptation and regeneration. Prolonged inflammation drives fibrogenic remodeling of the liver. Recently, considerable advances in the development of *in vivo* and *in vitro* models of NASH have been made ^14– 16^. The criteria for the validation of such models as models of human NASH are clearer ^17^. In reviewing data from such models for understanding human disease, it is important to ascertain if such validations have been performed in the model. These criteria include whether gene manipulations that are not reflective of the human state are used, if the macronutrient composition reflects human diet, if features commonly seen in humans such as obesity, systemic inflammation, dyslipidemia, insulin resistance, steatohepatitis, and fibrosis are present, and if there is concordance of cell signaling and transcriptome to human disease.

## Natural history and outcome of non-alcoholic fatty liver disease

Most of the data on the natural history of NAFLD are based on indirect evidence and have come from a selected population. Despite NAFLD’s high prevalence, only a minority of NAFLD patients progress to significant fibrosis or experience associated morbidity ^18^. The reason for this variability is, partly, subtle individual genetic differences that change one’s response to environmental factors and lifestyle, thus determining disease phenotype ^19– 21^. Recent data from a population study in Olmsted County reported a significantly higher mortality in NAFLD patients compared with the general population; however, overall mortality and liver-related death were lower than previously reported from referral centers. Mortality in these patients was associated with older age, glucose intolerance/diabetes, and the presence of cirrhosis. The top three leading causes of death in patients with NAFLD in descending order are cardiovascular disease, cancer, and liver disease ^22^. A patient’s chance of progressing to advanced liver disease, including hepatic decompensation and HCC, is higher if they suffer from NASH than if they suffer from NAFLD. Recent studies have suggested that NAFLD can evolve to NASH with advanced fibrosis, which would imply that it may not be an entirely benign condition ^23, 24^. A recent study found that 44% of patients with NAFLD at the index liver biopsy progressed to NASH and 37% progressed to fibrosis, including 22% to advanced degree ^25^. Managing NAFLD requires markedly increased healthcare resources as fibrosis worsens, especially after the development of cirrhosis. Although there are no exact models to estimate the incidence and the disease burden of NAFLD in the next few years, the changing trends of obesity and DM suggest that this problem is increasing worldwide and might place a growing strain on healthcare systems.

## Diagnosis

NAFLD remains asymptomatic in a significant proportion of patients, and the diagnosis is often suspected when liver functions are found to be abnormal on biochemical testing or hepatic imaging (ultrasonography, CT, or MRI of liver) suggests fatty liver when performed for some other reason. Liver biopsy remains the gold standard for diagnostic evaluation of NAFLD. In the past, the NASH Clinical Research Network histological scoring system was the most widely used, representing a validated scoring system that generates a NAFLD activity score (NAS). A NAS of 5 or more is sometimes considered NASH and less than 3 is considered not NASH ^26^. However, NAS cannot be used as a surrogate for discrimination between NASH and NAFLD, although it is useful for the histological diagnosis ^27, 28^. Given that the prevalence of NAFLD is high, using liver biopsy to detect fibrosis-cirrhosis is unfeasible. The accuracy of liver biopsy to assess fibrosis has also been questioned owing to sampling errors and intra- and inter-observer variability that may lead to over- or under-staging. Cost, procedure-related complications, and intra- and inter-observer variations in reporting the histology are the major drawbacks of liver biopsy, and, therefore, it is usually not recommended in clinical practice, except in circumstances where other differential diagnoses are to be excluded. Over the past decade, there has been a growing interest in alternative novel strategies for the non-invasive evaluation of fibrosis. These techniques require two distinct but complementary approaches: the measurement of serum biomarkers or the estimation of liver stiffness using ultrasound-based elastography with transient elastography (TE) as the forerunner ^29^. In NAFLD patients, TE, the fibrosis index (FIB-4), and NAFLD fibrosis scores are the best validated non-invasive tests ^30, 31^. For instance, in a recent meta-analysis ^32^ based on 64 studies including a total of 13,046 NAFLD patients, the summary AUROC values of TE, FIB-4, and the NAFLD fibrosis score for diagnosing severe fibrosis-cirrhosis were 0.88, 0.84, and 0.84, respectively. When all three were compared head to head, TE was the most accurate in the diagnosis of cirrhosis ^33^. Lastly, recent evidence shows that non-invasive tests, including FIB-4 and NAFLD fibrosis score as well as TE-derived liver stiffness measurements, accurately identify the subgroup of patients with NAFLD at a higher risk of developing liver-related complications and death or liver transplantation ^34^. Liver enzymes can often be normal in a number of patients with NAFLD. Although several biochemical markers, such as TNF-a, IL-6, CRP, pentraxin, ferritin, serum prolidase enzyme activity, soluble receptor for advanced glycation end product, and cytokeratin-18, have been proposed as useful in predicting the severity of NAFLD/NASH in the past, none of these markers have shown sufficient sensitivity or specificity for routine clinical application for diagnosis ^35^. Ultrasonography, CT, and MRI of the liver are the standard imaging modalities used in clinical practice for the diagnosis of NAFLD. In general, about 30% of liver steatosis cases should be present for sonography to detect NAFLD. TE is an ultrasound-based imaging technique to detect the degree of fibrosis in patients with NAFLD and NASH. The sensitivity and specificity of TE to diagnose various stages of fibrosis have been reported to be 79–92% and 75–92%, respectively ^36^. Recent evidence also suggests that the ultrasound-based controlled attenuation parameter value used in the TE technique can predict the degree of steatosis in patients with NAFLD ^37^. The gold standard for the non-invasive assessment of hepatic steatosis is the use of MRI protein density fat fraction. Newer MRI techniques, such as MR elastography, can stage the degree of fibrosis non-invasively to diagnose and assess the prognosis of patients with NAFLD ^38^.

## Treatment

**Figure 1.**
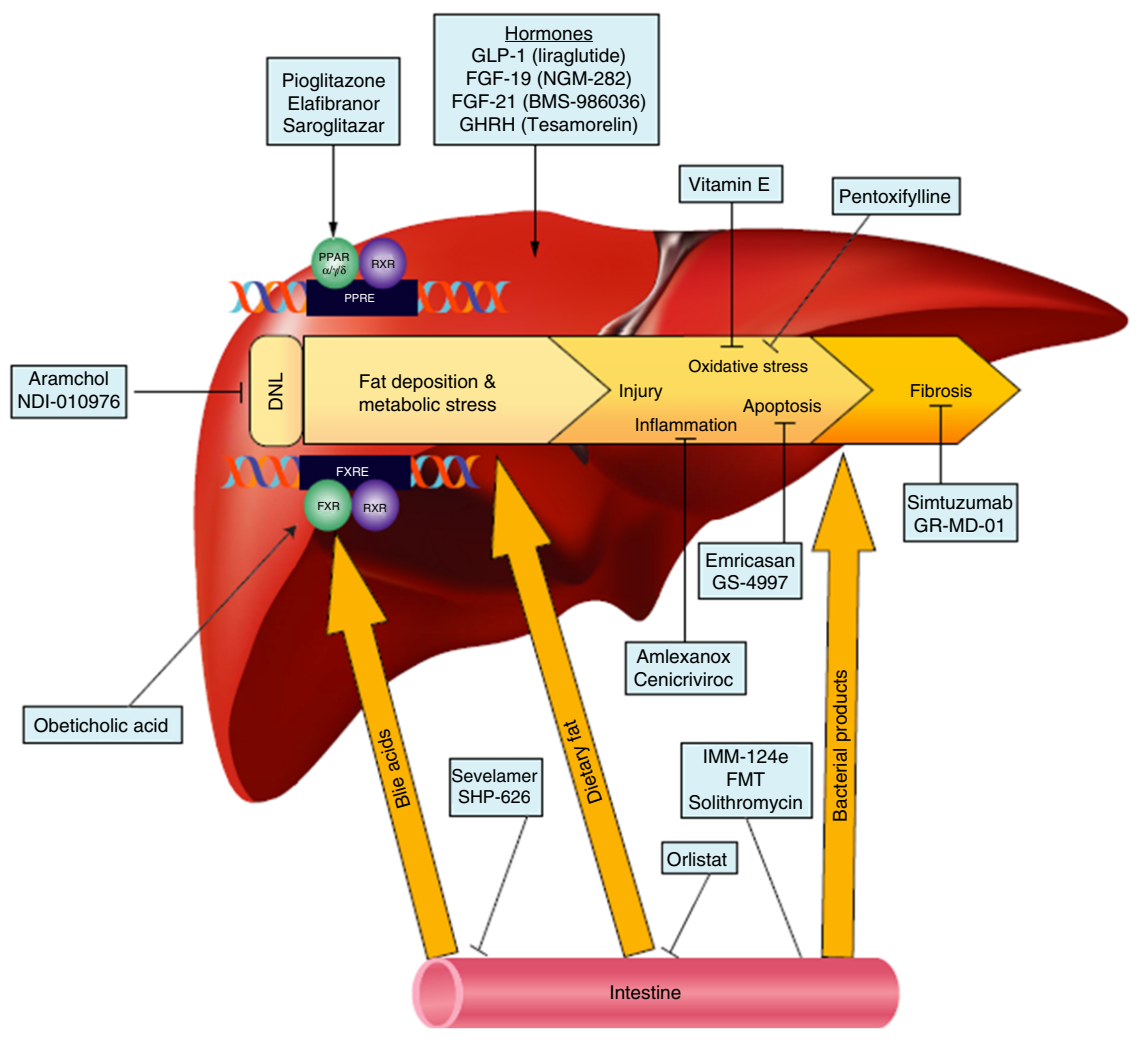
Targets of upcoming therapies for non-alcoholic fatty liver disease (NAFLD). DNL, *de novo* lipogenesis; FGF, fibroblast growth factor; FMT, fecal microbial transplant; FXR, farnesoid X receptor; FXRE, FXR response element; GHRH, growth hormone-releasing hormone; GLP-1, glucagon-like peptide-1; PPAR, peroxisome proliferator-activated receptor; PPRE, PPAR response element; RXR, retinoid X receptor. Reprinted with permission from Rotman Y and Sanyal AJ. *Current and upcoming pharmacotherapy for non-alcoholic fatty liver disease*. Gut. 2017;66:180–190.

Lifestyle modification, consisting of diet and exercise, is the cornerstone of therapy for NAFLD and has been shown by many studies to improve liver histology ^39, 40^. However, lifestyle modification is difficult to achieve and to sustain ^41^. The remarkable progress that has been made in previous years in understanding disease pathogenesis has led to an explosion of medical therapies targeting various aspects of the fat accumulation and injury pathways. These therapies can be classified according to their intended targets into four general groups ( Figure 1).

### 1. Targeting metabolism and oxidative stress

***Antioxidants***. Large randomized clinical trials have proven the beneficial effects of vitamin E in patients with NASH ( Table 1). In an innovative randomized double-blind placebo-controlled trial (RDBPCT), the authors found that a daily dose of 800 IU of vitamin E for 96 weeks improved the histological features of NASH (hepatic steatosis, lobular inflammation, and hepatocellular ballooning) in approximately 43% of non-diabetic patients compared with 19% of placebo ( *P* = 0.001) ^42^. In an analysis conducted recently of patients from the control group of the FLINT trial, vitamin E’s ability to improve NASH histological features was further confirmed ^43, 44^.

**Table 1.** Clinical trials of medications for non-alcoholic fatty liver disease (NAFLD).

Medication	Mechanism	Current status	Study identifier
Pioglitazone	PPARγ agonist	Phase IV (completed)	NCT00994682
Elafibranor	PPARα/δ agonist	Phase III (recruiting)	NCT02704403
Saroglitazar	PPARα/γ agonist	Phase II (recruiting)	NCT03061721
Obeticholic acid	FXR agonist	Phase III (recruiting)	NCT03439254
Liraglutide	GLP-1 receptor agonist	Phase IV (completed)	NCT02147925
Aramchol	SCD inhibitor	Phase II (recruiting)	NCT02684591
Volixibat (SHP-626)	ASBT inhibitor	Phase II (recruiting)	NCT02787304
BMS-986036	FGF-21 analogue	Phase II (not yet recruiting)	NCT03486899
NGM-282	FGF-19 analogue	Phase II (recruiting)	NCT02443116
Tesamorelin	GHRH analogue	Phase II (not yet recruiting)	NCT03375788
NDI-010976	ACC inhibitor	Phase I (completed)	
GS-9674	FXR agonist	Phase I (recruiting)	NCT02808312
Dur-928	Sulfated oxysterol	Phase I	
AZD4076	miR-103/-107 antagonist	Phase I (active, not recruiting)	NCT02612662
Rosuvastatin	HMG-CoA reductase inhibitor	Phase IV (not yet recruiting)	NCT03434613
INT-767	FXR/TGR5 agonist	Preclinical	
Sevelamer	Bile acid sequestrant	Preclinical	
Vitamin E	Anti-oxidant	Phase II (active, not recruiting)	NCT01792115
Pentoxifylline	PDE inhibitor	Phase II (completed)	NCT02283710
Cenicriviroc	CCR2/CCR5 antagonist	Phase II (enrolling by invitation)	NCT03059446
Emricasan	Caspase inhibitors	Phase II (recruiting)	NCT03205345
GS-4997	ASK1 inhibitor	Phase III (active, not recruiting)	NCT03053050
Amlexanox	IKKε/TBK1 inhibitor	Phase II (active, not recruiting)	NCT01975935
PXS-4728A	VAP-1 inhibitor	Phase I (completed)	
Orlistat	Intestinal lipase inhibitor	Phase IV (completed)	NCT00160407
IMM-124e	IgG-rich bovine colostrum	Phase II (recruiting)	NCT03042767
Solithromycin	Antibiotic	Phase II (completed)	NCT02510599
Fecal microbial transplant	Modulation of gut microbiome	Phase II (active, not recruiting)	NCT02496390
Simtuzumab	LOXL2 antibody	Phase II (completed)	NCT02466516
GR-MD-02	Galectin-3 inhibitor	Phase II (completed)	NCT02421094

ACC, acetyl-CoA carboxylase; ASBT, apical sodium-dependent bile acid transporter; ASK1, apoptosis signal-regulating kinase 1; CCR, C-C chemokine receptor; GLP, glucagon-like peptide; FGF, fibroblast growth factor; FXR, farnesoid X receptor; GHRH, growth hormone-releasing hormone; HMG-CoA, 3-hydroxy-3-methyl-glutaryl-coenzyme A; LOXL2, lysyl oxidase-like 2; miR, microRNA; PDE, phosphodiesterase; PPAR, peroxisome proliferator-activator receptor; SCD, stearoyl CoA desaturase; TBK1, TANK-binding kinase 1; VAP, vascular adhesion protein.

***Lipid-lowering agents***. A recent study demonstrated the underutilization of statins in patients with NAFLD ^45^ ( Table 1). A prospective trial in 20 patients with biopsy-proven NASH and dyslipidemia determined the effect of 12 months of rosuvastatin (10 mg/day) on liver histology. In total, 19 out of the 20 patients enrolled demonstrated complete resolution of NASH despite no change in weight compared with baseline ^46^. Aramchol is a synthetic lipid that inhibits the stearoyl coenzyme A desaturase 1, a key enzyme in lipid metabolism ^47^. A recent phase II RDBPCT compared two doses of aramchol (100 mg and 300 mg, daily) to placebo for three months in 60 patients with biopsy-proven NAFLD including six with NASH. At the end of the trial, hepatic fat was significantly reduced, as measured by magnetic resonance spectroscopy, in patients who were treated with 100 mg daily aramchol compared to those given placebo (12.57% versus 6.39%, respectively; *P* = 0.02) ^48^.

***Targeting insulin resistance***

**Bile-acid-based insulin sensitization**

Farnesoid X receptor (FXR) is a nuclear bile acid receptor which inhibits further bile acid production when activated via the rate-limiting enzyme cholesterol 7 alpha-hydroxylase (CYP7A1) ( Table 1). A recent study demonstrated that there are changes in the bile acid composition in those with NASH ^49^. There is a progressive increase in primary bile acids and a decrease in secondary bile acids, which would be predicted to reduce FXR effects.

Obeticholic acid (OCA) is a selective FXR agonist that showed promising anti-inflammatory and anti-fibrotic effects in animal studies ^50^. Its potential has been realized in the FLINT trial, where it was shown to be superior to placebo for the reduction of disease activity as well as fibrosis. It also increases LDL cholesterol and causes pruritus in up to 20% of individuals. This has led to efforts to develop small molecules as FXR agonists that do not have these adverse effects. Several such compounds are in early phase trials. It is also proposed that binding of FXR by bile acids in the intestine releases FGF19, which also ameliorates NASH; a recent early phase trial demonstrated rapid de-fatting of the liver with FGF19. On the other hand, blocking bile acid re-absorption in the ileum also depletes the bile acid pool and pulls cholesterol into bile acid synthesis, decreasing the cholesterol load. Further delivery of bile acids to the ileum can release GLP1, which has insulin-sensitizing properties. The use of bile acid transport inhibitors is being tested in clinical trials.

Recently, a number of additional bile acid receptors have been proposed as targets for drug development in NASH. TGR5 is a receptor with important functions and effects on hepatic lipid regulation and glucose metabolism. INT-767 is an investigation drug which activates both FXR and TGR5 and is being studied currently in phase IIb RDBPCT in patients with NASH (NCT02854605).

***Peroxisome proliferator-activator receptors***. Recently, a peroxisome proliferator-activated receptor (PPAR) δ agonist, MBX-8025, was shown to abolish lipotoxicity and ameliorate NASH in a diabetic mouse model ^51^. Elafibranor (GFT-505) is a dual PPARα/δ agonist which has been shown to improve liver, adipose tissue, and peripheral tissue insulin sensitivity and reduce alanine aminotransferase (ALT) levels in patients with metabolic syndrome ^52^. Thiazolidinediones, including pioglitazone, are PPARγ agonists used in the treatment of diabetes and have been demonstrated to be effective in NASH ^53^.

The glitazars are dual PPARα/γ agonists which aim to combine the beneficial effects of activating both PPAR receptors. In a mouse model of NASH, saroglitazar was found to reduce steatosis and ALT as well as improve liver histology ^54^. A subsequent retrospective study of NAFLD patients with dyslipidemia treated with saroglitazar for 24 weeks showed a significant decrease in ALT compared with baseline ^55^. A phase II open-label study (PRESS VIII) evaluated the effectiveness of saroglitazar among 32 patients with biopsy-proven NASH ^54^. After 12 weeks of treatment, a 52% decrease in ALT was shown. A phase III RDBPCT is currently ongoing in India to assess the effect of saroglitazar versus placebo for 52 weeks in biopsy-proven non-cirrhotic NASH (Clinical Trials Registry-India CTRI/2015/10/006236) ( Table 1).

***Metformin***. A recent meta-analysis has shown that metformin leads to normalization of serum aminotransferases in a significantly greater proportion of patients when compared to dietary changes, and it also improved steatosis on imaging ^56^.

***Incretins and sodium–glucose cotransporter 2 inhibitors***. Recently, a multicenter RDBPCT evaluated liraglutide in 52 subjects with NASH. After 48 weeks of treatment, NASH resolved in 39% of patients treated with subcutaneous liraglutide injections compared to only 9% in the placebo group (relative risk 4.3 [95% CI: 1.0–17.7]; *P* = 0.019). Additionally, two patients on liraglutide (9%) versus eight patients (36%) on placebo exhibited fibrosis progression ^57^. The newest class of diabetic medications on the market are sodium–glucose cotransporter 2 (SGLT-2) inhibitors. A recent retrospective study evaluated the effectiveness of SGLT-2 inhibitor ipragliflozin (50 mg/day) on liver enzymes and FIB-4 in 50 diabetic NAFLD patients ^58^. Over an average follow-up of 451 days, there was a significant decrease in body weight, ALT, and FIB-4 compared with baseline values ( Table 1).

***FGF21***. This is a hepatokine with insulin-sensitizing and anti-fibrotic properties. In a phase IIa study, daily as well as weekly dosing of FGF21 was associated with improvement in hepatic steatosis and a decrease in hepatic stiffness.

### 2. Targeting inflammation

Cenicriviroc (CVC) is an oral dual antagonist of C-C chemokine receptors (CCR2/CCR5) that was recently studied in a phase IIb RDBPCT, the CENTAUR trial. The trial showed improvement of fibrosis by at least one stage ( *P* = 0.023) and a decrease in IL-6, high-sensitivity CRP, and fibrinogen levels in patients treated with CVC compared to placebo. A phase IIa study of cenicriviroc (ORION) aiming to assess the effect of 24 weeks of treatment on insulin sensitivity, liver enzymes, and liver imaging in obese patients with insulin resistance and suspected NAFLD in HIV-infected subjects provided encouraging initial data (NCT02330549). The CENTAUR study by Friedman *et al*. evaluated the effect of cenicriviroc on the histology of NASH ^59^; this showed improved biochemical evidence of inflammation and histological evidence of decreased fibrosis. However, it did not affect the presence of steatohepatitis or disease activity as assessed by histological assessment.

Another investigational anti-inflammatory agent is amlexanox; it functions through different mechanisms by inhibiting NFκB-pathway-produced IKKε kinases and TANK-binding kinase 1 (TBK1). This drug, currently used for the treatment of recurrent aphthous ulcers and asthma, is being studied in a phase II RDBPCT in obese patients with type 2 diabetes and NAFLD (NCT01975935) ( Table 1).

### Targeting apoptosis

Emricasan is a first-in-class caspase protease inhibitor that has been studied in preclinical settings. In a recent phase II RDBPCT of 38 study participants with non-cirrhotic NAFLD, 28 days of emricasan (25 mg twice daily) resulted in a substantial decrease in liver enzymes and cytokeratin 18 fragments, a surrogate of liver apoptosis ^60^. A phase IIb trial of emricasan versus placebo (ENCORE-NF) is currently ongoing to evaluate the efficacy of 72 weeks of emricasan (10 mg per day or 100 mg per day) in patients with biopsy-proven NASH. The primary outcome is improvement in fibrosis without worsening of NASH (NCT02686762). Inhibition of apoptosis signal-regulating kinase (ASK1) reduces liver steatosis and fibrosis in a murine model of diet-induced NASH ^61^. An open-label phase II trial in 72 NASH patients with stage 2/3 fibrosis randomized to an oral ASK1 inhibitor, selonsertib (formerly called GS-4997; 6 mg or 18 mg/day), versus lysyl oxidase-like 2 antibody simtuzumab (25 mg subcutaneous weekly) versus selonsertib and simtuzumab was recently completed ^62^. Preliminary analysis showed that patients who received selonsertib (with or without simtuzumab) were more likely to demonstrate decreased hepatic steatosis, decreased fibrotic stage, and at least 15% decrease in liver stiffness on magnetic resonance elastography compared with simtuzumab alone. Anti-steatotic and anti-fibrotic effects of selonsertib were dose dependent ^63^ ( Table 1).

### 3. Targeting fibrosis

The lysyl oxidase-like 2 antibody simtuzumab is currently in a phase IIb trial in NASH patients with advanced fibrosis but without cirrhosis (NCT01672866) ( Table 1). Study participants are randomized to biweekly subcutaneous injections of simtuzumab (75 or 120 mg) versus placebo for 96 weeks, followed by an additional 240 weeks of open-label phase. Simtuzumab is also being investigated in a phase IIb trial in NASH patients with compensated cirrhosis (NCT01672879). The primary end point being assessed is mean change in hepatic venous pressure gradient as well as event-free survival. Galectin-3 is a member of a family of proteins that bind to terminal galactose residues on glycoprotein and is found to be increased during acute or chronic inflammation resulting in fibrogenesis. GR-MD-02 is a galectin-3 inhibitor that was shown to improve NASH fibrosis in animal studies ^63^ and is now being evaluated in two phase II RDBPCTs in patients with NASH fibrosis and NASH cirrhosis, one of which has already completed enrollment (NCT02462967, NCT02421094). The final results of a phase II trial indicated that a subset of individuals with mild portal hypertension improved on galectin.

### 4. Targeting the gut

***Microbiome-based therapies***. Bovine colostrum is enriched with IgG directed against antigens injected into cows immediately prior to calving. An IgG-rich bovine colostrum extract, IMM-124e, generated from cows immunized against lipopolysaccharide was shown to improve insulin sensitivity, glycemic control, and liver enzymes in a small pilot study ^64^. A phase II trial is currently evaluating 24 weeks of IMM-124e on biopsy-proven NASH (ClinicalTrials.gov identifier NCT02316717). Solithromycin, a macrolide antibiotic with anti-inflammatory properties, has been found to improve NASH in animal studies ^65^ and is currently being studied in a phase II clinical trial (NCT02510599) ( Table 1).

### Anti-obesity medications

A small pilot study suggested that Orlistat-mediated weight loss is associated with reduction in hepatic steatosis ^66^. However, the impact of Orlistat on steatohepatitis and its ability to slow the progression of NASH to cirrhosis remains unknown ( Table 1).

## Summary

NAFLD is the most common cause of chronic liver disease in the Western world today. With rising levels of obesity and type 2 DM, its prevalence will increase in the future and cause considerable morbidity and mortality. The field of NAFLD continues to evolve rapidly. Advances in the understanding of classical steatosis and disease progression are also reviewed with a view toward providing translational insights into how this knowledge can be used to prevent or treat the disease in the future. Despite considerable research and multiple clinical trials, at present no single pharmacologic agent has achieved a clinically meaningful benefit/risk profile to warrant regulatory approval for marketing. There are currently a number of drugs undergoing pivotal trials as potential therapy for NASH. It is anticipated that the first drugs to be approved for NASH will likely become available by 2020. The ideal treatment will lead, in the short term, to a reduction in liver inflammation and fibrosis as well as an improvement in insulin sensitivity and metabolic complications but, in the long term, will need to reduce cardiovascular and liver outcomes.

## References

[1] Perumpail BJ Khan MA Yoo ER: Clinical epidemiology and disease burden of nonalcoholic fatty liver disease. *World J Gastroenterol.* 2017;23(47):8263–76. 10.3748/wjg.v23.i47.8263 29307986 PMC5743497

[2] Welsh JA Karpen S Vos MB: Increasing prevalence of nonalcoholic fatty liver disease among United States adolescents, 1988-1994 to 2007-2010. *J Pediatr.* 2013;162(3):496–500.e1. 10.1016/j.jpeds.2012.08.043 23084707 PMC3649872

[3] Eguchi Y Hyogo H Ono M: Prevalence and associated metabolic factors of nonalcoholic fatty liver disease in the general population from 2009 to 2010 in Japan: a multicenter large retrospective study. *J Gastroenterol.* 2012;47(5):586–95. 10.1007/s00535-012-0533-z 22328022

[4] Fazel Y Koenig AB Sayiner M: Epidemiology and natural history of non-alcoholic fatty liver disease. *Metabolism.* 2016;65(8):1017–25. 10.1016/j.metabol.2016.01.012 26997539

[5] Bedogni G Miglioli L Masutti F: Prevalence of and risk factors for nonalcoholic fatty liver disease: the Dionysos nutrition and liver study. *Hepatology.* 2005;42(1):44–52. 10.1002/hep.20734 15895401

[6] Younossi Z Anstee QM Marietti M: Global burden of NAFLD and NASH: trends, predictions, risk factors and prevention. *Nat Rev Gastroenterol Hepatol.* 2018;15(1):11–20. 10.1038/nrgastro.2017.109 28930295

[7] Younossi ZM Koenig AB Abdelatif D: Global epidemiology of nonalcoholic fatty liver disease-Meta-analytic assessment of prevalence, incidence, and outcomes. *Hepatology.* 2016;64(1):73–84. 10.1002/hep.28431 26707365

[8] Browning JD Szczepaniak LS Dobbins R: Prevalence of hepatic steatosis in an urban population in the United States: impact of ethnicity. *Hepatology.* 2004;40(6):1387–95. 10.1002/hep.20466 15565570

[9] Estes C Razavi H Loomba R: Modeling the epidemic of nonalcoholic fatty liver disease demonstrates an exponential increase in burden of disease. *Hepatology.* 2018;67(1):123–33. 10.1002/hep.29466 28802062 PMC5767767

[10] Younossi ZM Blissett D Blissett R: The economic and clinical burden of nonalcoholic fatty liver disease in the United States and Europe. *Hepatology.* 2016;64(5):1577–86. 10.1002/hep.28785 27543837

[11] Martini EM Garrett N Lindquist T: The boomers are coming: a total cost of care model of the impact of population aging on health care costs in the United States by Major Practice Category. *Health Serv Res.* 2007;42(1 Pt 1):201–18. 10.1111/j.1475-6773.2006.00607.x 17355589 PMC1955745

[12] Younossi ZM Henry L: Economic and Quality-of-Life Implications of Non-Alcoholic Fatty Liver Disease. *Pharmacoeconomics.* 2015;33(12):1245–53. 10.1007/s40273-015-0316-5 26233836

[13] Perla FM Prelati M Lavorato M: The Role of Lipid and Lipoprotein Metabolism in Non-Alcoholic Fatty Liver Disease. *Children (Basel).* 2017;4(6): pii: E46. 10.3390/children4060046 28587303 PMC5483621

[14] Caldwell SH: Recent Advances in the Treatment of NASH. *Gastroenterol Hepatol (N Y).* 2006;2(1):29–31. 28210193 PMC5307258

[15] Kanuri G Bergheim I: *In vitro* and *in vivo* models of non-alcoholic fatty liver disease (NAFLD). *Int J Mol Sci.* 2013;14(6):11963–80. 10.3390/ijms140611963 23739675 PMC3709766

[16] Feaver RE Cole BK Lawson MJ: Development of an *in vitro* human liver system for interrogating nonalcoholic steatohepatitis. *JCI Insight.* 2016;1(20):e90954. 10.1172/jci.insight.90954 27942596 PMC5135271

[17] Morrison MC Kleemann R van Koppen A: Key Inflammatory Processes in Human NASH Are Reflected in Ldlr ^-/-^.Leiden Mice: A Translational Gene Profiling Study. *Front Physiol.* 2018;9:132. 10.3389/fphys.2018.00132 29527177 PMC5829089

[18] Anstee QM Targher G Day CP: Progression of NAFLD to diabetes mellitus, cardiovascular disease or cirrhosis. *Nat Rev Gastroenterol Hepatol.* 2013;10(6):330–44. 10.1038/nrgastro.2013.41 23507799

[19] Anstee QM Day CP: The genetics of NAFLD. *Nat Rev Gastroenterol Hepatol.* 2013;10(11):645–55. 10.1038/nrgastro.2013.182 24061205

[20] Valenti L Al-Serri A Daly AK: Homozygosity for the patatin-like phospholipase-3/adiponutrin I148M polymorphism influences liver fibrosis in patients with nonalcoholic fatty liver disease. *Hepatology.* 2010;51(4):1209–17. 10.1002/hep.23622 20373368

[21] Liu YL Reeves HL Burt AD: *TM6SF2* rs58542926 influences hepatic fibrosis progression in patients with non-alcoholic fatty liver disease. *Nat Commun.* 2014;5:4309. 10.1038/ncomms5309 24978903 PMC4279183

[22] Adams LA Lymp JF St Sauver J: The natural history of nonalcoholic fatty liver disease: a population-based cohort study. *Gastroenterology.* 2005;129(1):113–21. 10.1053/j.gastro.2005.04.014 16012941

[23] Wong VW Wong GL Choi PC: Disease progression of non-alcoholic fatty liver disease: a prospective study with paired liver biopsies at 3 years. *Gut.* 2010;59(7):969–74. 10.1136/gut.2009.205088 20581244

[24] Pais R Charlotte F Fedchuk L: A systematic review of follow-up biopsies reveals disease progression in patients with non-alcoholic fatty liver. *J Hepatol.* 2013;59(3):550–6. 10.1016/j.jhep.2013.04.027 23665288

[25] McPherson S Hardy T Henderson E: Evidence of NAFLD progression from steatosis to fibrosing-steatohepatitis using paired biopsies: implications for prognosis and clinical management. *J Hepatol. * 2015;62(5):1148–55. 10.1016/j.jhep.2014.11.034 25477264

[26] Kleiner DE Brunt EM Van Natta M: Design and validation of a histological scoring system for nonalcoholic fatty liver disease. *Hepatology.* 2005;41(6):1313–21. 10.1002/hep.20701 15915461

[27] Brunt EM Kleiner DE Wilson LA: Nonalcoholic fatty liver disease (NAFLD) activity score and the histopathologic diagnosis in NAFLD: distinct clinicopathologic meanings. *Hepatology.* 2011;53(3):810–20. 10.1002/hep.24127 21319198 PMC3079483

[28] Brunt EM Kleiner DE Behling C: Misuse of scoring systems. *Hepatology.* 2011;54(1):369–70, author reply 370-1. 10.1002/hep.24347 21488072

[29] Castera L Pinzani M: Non-invasive assessment of liver fibrosis: are we ready? *Lancet.* 2010;375(9724):1419–20. 10.1016/S0140-6736(09)62195-4 20417845

[30] Castera L: Noninvasive Evaluation of Nonalcoholic Fatty Liver Disease. *Semin Liver Dis.* 2015;35(3):291–303. 10.1055/s-0035-1562948 26378645

[31] Friedrich-Rust M Poynard T Castera L: Critical comparison of elastography methods to assess chronic liver disease. *Nat Rev Gastroenterol Hepatol.* 2016;13(7):402–11. 10.1038/nrgastro.2016.86 27273167

[32] Xiao G Zhu S Xiao X: Comparison of laboratory tests, ultrasound, or magnetic resonance elastography to detect fibrosis in patients with nonalcoholic fatty liver disease: A meta-analysis. *Hepatology.* 2017;66(5):1486–501. 10.1002/hep.29302 28586172

[33] Boursier J Vergniol J Guillet A: Diagnostic accuracy and prognostic significance of blood fibrosis tests and liver stiffness measurement by FibroScan in non-alcoholic fatty liver disease. *J Hepatol.* 2016;65(3):570–8. 10.1016/j.jhep.2016.04.023 27151181

[34] Angulo P Bugianesi E Bjornsson ES: Simple noninvasive systems predict long-term outcomes of patients with nonalcoholic fatty liver disease. *Gastroenterology.* 2013;145(4):782–9.e4. 10.1053/j.gastro.2013.06.057 23860502 PMC3931256

[35] Oh H Jun DW Saeed WK: Non-alcoholic fatty liver diseases: update on the challenge of diagnosis and treatment. *Clin Mol Hepatol.* 2016;22(3):327–35. 10.3350/cmh.2016.0049 27729634 PMC5066376

[36] Kwok R Tse YK Wong GL: Systematic review with meta-analysis: non-invasive assessment of non-alcoholic fatty liver disease--the role of transient elastography and plasma cytokeratin-18 fragments. *Aliment Pharmacol Ther.* 2014;39(3):254–69. 10.1111/apt.12569 24308774

[37] Karlas T Petroff D Sasso M: Individual patient data meta-analysis of controlled attenuation parameter (CAP) technology for assessing steatosis. *J Hepatol.* 2017;66(5):1022–30. 10.1016/j.jhep.2016.12.022 28039099

[38] Kinner S Reeder SB Yokoo T: Quantitative Imaging Biomarkers of NAFLD. *Dig Dis Sci.* 2016;61(5):1337–47. 10.1007/s10620-016-4037-1 26848588 PMC4854639

[39] Wong VW Chan RS Wong GL: Community-based lifestyle modification programme for non-alcoholic fatty liver disease: a randomized controlled trial. *J Hepatol.* 2013;59(3):536–42. 10.1016/j.jhep.2013.04.013 23623998

[40] Vilar-Gomez E Martinez-Perez Y Calzadilla-Bertot L: Weight Loss Through Lifestyle Modification Significantly Reduces Features of Nonalcoholic Steatohepatitis. *Gastroenterology.* 2015;149(2):367–78.e5; quiz e14-5. 10.1053/j.gastro.2015.04.005 25865049

[41] Stewart KE Haller DL Sargeant C: Readiness for behaviour change in non-alcoholic fatty liver disease: implications for multidisciplinary care models. *Liver Int.* 2015;35(3):936–43. 10.1111/liv.12483 24521540 PMC4266620

[42] Sanyal AJ Chalasani N Kowdley KV: Pioglitazone, vitamin E, or placebo for nonalcoholic steatohepatitis. *N Engl J Med.* 2010;362(18):1675–85. 10.1056/NEJMoa0907929 20427778 PMC2928471

[43] Kowdley KV: Efficacy and safety of vitamin E in nonalcoholic steatohepatitis patients with and without diabetes: pooled analysis from the PIVENS and FLINT NIDDK NASH CRN trials. *Hepatology.* 2015;62:264A.

[44] Neuschwander-Tetri BA Brunt EM Wehmeier KR: Improved nonalcoholic steatohepatitis after 48 weeks of treatment with the PPAR-gamma ligand rosiglitazone. *Hepatology.* 2003;38(4):1008–17. 10.1053/jhep.2003.50420 14512888

[45] Blais P Lin M Kramer JR: Statins Are Underutilized in Patients with Nonalcoholic Fatty Liver Disease and Dyslipidemia. *Dig Dis Sci.* 2016;61(6):1714–20. 10.1007/s10620-015-4000-6 26707137

[46] Kargiotis K Athyros VG Giouleme O: Resolution of non-alcoholic steatohepatitis by rosuvastatin monotherapy in patients with metabolic syndrome. *World J Gastroenterol.* 2015;21(25):7860–8. 10.3748/wjg.v21.i25.7860 26167086 PMC4491973

[47] Dobrzyn P Dobrzyn A Miyazaki M: Stearoyl-CoA desaturase 1 deficiency increases fatty acid oxidation by activating AMP-activated protein kinase in liver. *Proc Natl Acad Sci U S A.* 2004;101(17):6409–14. 10.1073/pnas.0401627101 15096593 PMC404058

[48] Safadi R Konikoff FM Mahamid M: The fatty acid-bile acid conjugate Aramchol reduces liver fat content in patients with nonalcoholic fatty liver disease. *Clin Gastroenterol Hepatol.* 2014;12(12):2085–91.e1. 10.1016/j.cgh.2014.04.038 24815326

[49] Puri P Daita K Joyce A: The presence and severity of nonalcoholic steatohepatitis is associated with specific changes in circulating bile acids. *Hepatology.* 2017. 10.1002/hep.29359 28696585 PMC5764808

[50] Cipriani S Mencarelli A Palladino G: FXR activation reverses insulin resistance and lipid abnormalities and protects against liver steatosis in Zucker (fa/fa) obese rats. *J Lipid Res.* 2010;51(4):771–84. 10.1194/jlr.M001602 19783811 PMC2842143

[51] Haczeyni F: PPAR-δ agonist MBX-8025 abolishes lipotoxicity and reverses NASH in diabetic obese mice. *Hepatology.* 2016;64:129A.

[52] Cariou B Hanf R Lambert-Porcheron S: Dual peroxisome proliferator-activated receptor α/δ agonist GFT505 improves hepatic and peripheral insulin sensitivity in abdominally obese subjects. *Diabetes Care.* 2013;36(10):2923–30. 10.2337/dc12-2012 23715754 PMC3781493

[53] Boettcher E Csako G Pucino F: Meta-analysis: pioglitazone improves liver histology and fibrosis in patients with non-alcoholic steatohepatitis. *Aliment Pharmacol Ther.* 2012;35(1):66–75. 10.1111/j.1365-2036.2011.04912.x 22050199 PMC3488596

[54] Jain MR Giri SR Trivedi C: Saroglitazar, a novel PPARα/γ agonist with predominant PPARα activity, shows lipid-lowering and insulin-sensitizing effects in preclinical models. *Pharmacol Res Perspect.* 2015;3(3):e00136. 10.1002/prp2.136 26171220 PMC4492752

[55] Saboo B: To Assess the Effect of 4 mg Saroglitazar on patients of diabetes dyslipidemia with nonalcoholic fatty liver disease. *Diabetes.* 2015;64:A180-A.

[56] Angelico F Burattin M Alessandri C: Drugs improving insulin resistance for non-alcoholic fatty liver disease and/or non-alcoholic steatohepatitis. *Cochrane Database Syst Rev.* 2007; (1):CD005166. 10.1002/14651858.CD005166.pub2 17253544

[57] Armstrong MJ Gaunt P Aithal GP: Liraglutide safety and efficacy in patients with non-alcoholic steatohepatitis (LEAN): a multicentre, double-blind, randomised, placebo-controlled phase 2 study. *Lancet.* 2016;387(10019):679–90. 10.1016/S0140-6736(15)00803-X 26608256

[58] Ohki T: SGLT-2 inhibitors improved liver inflammation and fibrosis of NAFLD patients with type 2 diabetes mellitus with a favorable effect of weight reduction. *Hepatology.* 2016;64:582A.26999257

[59] Friedman SL Ratziu V Harrison SA: A randomized, placebo-controlled trial of cenicriviroc for treatment of nonalcoholic steatohepatitis with fibrosis. *Hepatology.* 2018;67(5):1754–67. 10.1002/hep.29477 28833331 PMC5947654

[60] Shiffman M Freilich B Vuppalanchi R: LP37: A Placebo-controlled, multicenter, double-blind, randomised trial of emricasan in subjects with non-alcoholic fatty liver disease (Nafld) and raised transaminases. *J Hepatol.* 2015;62(Supplement 2):S282-S 10.1016/S0168-8278(15)30191-4

[61] Budas G Karnik S Jonnson T: Reduction of liver steatosis and fibrosis with an ask1 inhibitor in a murine model of NASH is accompanied by improvements in cholesterol, bile acid and lipid metabolism. *J Hepatol.* 2016;64(2):S170 10.1016/S0168-8278(16)01686-X

[62] Loomba R Lawitz E Mantry PS: GS-4997, an Inhibitor of Apoptosis Signal-Regulating Kinase (ASK1), Alone or in Combination with Simtuzumab for the Treatment of Nonalcoholic Steatohepatitis (NASH): A Randomized, Phase 2 Trial.2016;64(6):1119A Reference Source

[63] Reilly SM Chiang SH Decker SJ: An inhibitor of the protein kinases TBK1 and IKK-ɛ improves obesity-related metabolic dysfunctions in mice. *Nat Med.* 2013;19(3):313–21. 10.1038/nm.3082 23396211 PMC3594079

[64] Mizrahi M Shabat Y Ben Ya'acov A: Alleviation of insulin resistance and liver damage by oral administration of Imm124-E is mediated by increased Tregs and associated with increased serum GLP-1 and adiponectin: results of a phase I/II clinical trial in NASH. *J Inflamm Res.* 2012;5:141–50. 10.2147/JIR.S35227 23293533 PMC3534391

[65] Fernandes P: Mechanism of anti-NASH effects of Solithromycin in a predictive NASH HCC mouse model. *Hepatology.* 2015;62:1301A-A.

[66] Harrison SA Fecht W Brunt EM: Orlistat for overweight subjects with nonalcoholic steatohepatitis: A randomized, prospective trial. *Hepatology.* 2009;49(1):80–6. 10.1002/hep.22575 19053049

